# PLGA: the smart therapeutics goods carrier

**DOI:** 10.3389/fphar.2013.00020

**Published:** 2013-03-06

**Authors:** Radhika J. Poojari

**Affiliations:** Department of Biosciences and Bioengineering, Indian Institute of Technology Bombay (IIT B)Mumbai, India

**Figure 1:**
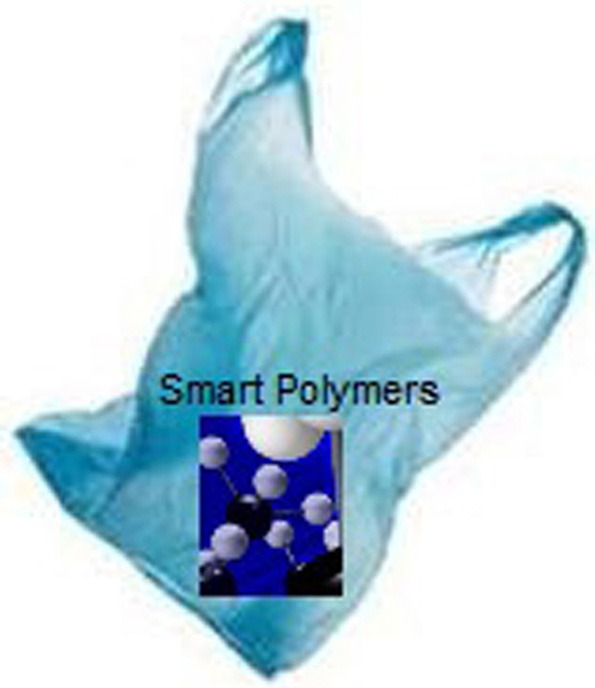
**Smart engineered PLGA polymers**.

Polymers have been used since decades as carriers for chemotherapeutic drugs. Among the various controlled release polymer technologies PLGA [poly(lactic acid-co-glycolic acid)] has the greatest clinical impact till date. Precisely engineered PLGA's is the need of the hour (Figure 1). For the first time, Elizabeth Enlow and colleagues have come out with a new strategy of fabricating PLGA via the PRINT technology.

Traditional methods of fabricating PLGA nanoparticles are emulsions, precipitations, spray drying, ultrasonication, and flow focusing. The effects of these process parameters on particle properties such as solvent, emulsifier, and particle composition affecting particle size, cargo release, and cargo encapsulation are the important issues. Poor encapsulation efficiency resulting in lower drug loadings limits the particles therapeutically effective dose is a challenge. So, is there a way out? Yes. The authors used the particle replication in non-wetting templates (PRINT) technology, a soft lithography platform based on a perfluorinated polyether elastomer which has the unique ability to fabricate particles of versatile sizes, shapes, and surface textures independent of process parameters. The PRINT process allows same particle geometry to be created with a variety of polymer molecular weights, polymer lactic acid to glycolic acid ratios, solvent systems, stabilizers, and cargos unlike the traditional techniques. The sizes and shapes can be used to affect cell uptake, biodistribution, and flow characteristics. Cargos can be easily encapsulated without process modifications. The authors have demonstrated high and efficient drug loadings up to 40% (w/w) with encapsulation efficiencies >90% with the PLGA PRINT nanoparticles of a potent chemotherapeutic drug Docetaxel. In comparison to this, maximum Docetaxel loading was 15% with encapsulation varying widely dependent on particular fabrication method and specific parameters used with the traditional methods (Enlow et al., 2011). The PRINT process has tight control over particle composition retaining the particle's physical characteristics, homogenous size, and shape, morphology as well as its consistency.

The authors tested the effectiveness of these PLGA PRINT nanoparticles by exposing them to Ovarian carcinoma cells, SKOV3 *in vitro*. The findings have shown higher toxicity with high encapsulation of Docetaxel which can be delivered intact to its desired cellular location in comparison to particles with lower drug loadings and the clinical formulation of Docetaxel, Taxotere. Further research is needed to establish its enhanced therapeutic potential *in vivo* at a lower dose and higher loadings.

The PRINT tailored PLGA drug delivery system is a very significant advance with the greater ability to encapsulate high therapeutic loadings efficiently, to protect cargo, program release kinetics, design multifunctionality, and compatibility beyond doubt. Therefore, developing this PRINT engineered polymeric PLGA is a goldmine as nano-carriers for chemotherapeutic drugs.
